# Antimalarial Activity of Potential Inhibitors of *Plasmodium falciparum* Lactate Dehydrogenase Enzyme Selected by Docking Studies

**DOI:** 10.1371/journal.pone.0021237

**Published:** 2011-07-14

**Authors:** Julia Penna-Coutinho, Wilian Augusto Cortopassi, Aline Alves Oliveira, Tanos Celmar Costa França, Antoniana Ursine Krettli

**Affiliations:** 1 Faculdade de Medicina, Universidade Federal de Minas Gerais, (UFMG), Belo Horizonte, Minas Gerais, Brazil; 2 Laboratório de Malária, Centro de Pesquisas René Rachou, FIOCRUZ, Belo Horizonte, Minas Gerais, Brazil; 3 Laboratory of Molecular Modeling Applied to the Chemical and Biological Defense (LMCBD), Military Institute of Engineering, Rio de Janeiro, Rio de Janeiro, Brazil; Federal University of São Paulo, Brazil

## Abstract

The *Plasmodium falciparum* lactate dehydrogenase enzyme (*Pf*LDH) has been considered as a potential molecular target for antimalarials due to this parasite's dependence on glycolysis for energy production. Because the LDH enzymes found in *P. vivax*, *P. malariae* and *P. ovale* (pLDH) all exhibit ∼90% identity to *Pf*LDH, it would be desirable to have new anti-pLDH drugs, particularly ones that are effective against *P. falciparum*, the most virulent species of human malaria. Our present work used docking studies to select potential inhibitors of pLDH, which were then tested for antimalarial activity against *P. falciparum in vitro* and *P. berghei* malaria in mice. A virtual screening in DrugBank for analogs of NADH (an essential cofactor to pLDH) and computational studies were undertaken, and the potential binding of the selected compounds to the *Pf*LDH active site was analyzed using Molegro Virtual Docker software. Fifty compounds were selected based on their similarity to NADH. The compounds with the best binding energies (itraconazole, atorvastatin and posaconazole) were tested against *P. falciparum* chloroquine-resistant blood parasites. All three compounds proved to be active in two immunoenzymatic assays performed in parallel using monoclonals specific to *Pf*LDH or a histidine rich protein (HRP2). The IC_50_ values for each drug in both tests were similar, were lowest for posaconazole (<5 µM) and were 40- and 100-fold less active than chloroquine. The compounds reduced *P. berghei* parasitemia in treated mice, in comparison to untreated controls; itraconazole was the least active compound. The results of these activity trials confirmed that molecular docking studies are an important strategy for discovering new antimalarial drugs. This approach is more practical and less expensive than discovering novel compounds that require studies on human toxicology, since these compounds are already commercially available and thus approved for human use.

## Introduction

Malaria is the most lethal parasitic disease in the world, annually affecting approximately 500 million people and resulting in 800,000 deaths, mostly in African sub-Saharan countries [1]. Brazil registered 306,000 cases of malaria in 2009, most of which were in the Amazonian region, as diagnosed and treated by Ministry of Health officers [2], [3]. Transmission occurs through the bite of *Anopheles* mosquitoes infected with the parasite and five different species may affect humans. *Plasmodium falciparum* is the most pathogenic species and may cause severe malaria and death in untreated nonimmune individuals, especially children under five [4].

The antimalarial treatment recommended for *P. falciparum* consists of drug combinations containing artemisinin derivatives (ACT) with other antimalarials, including quinoline compounds, such as amodiaquine and mefloquine. The quinolines act mainly by inhibiting hematin polymerization, thus intoxicating the parasite with the ferriprotoporphyrinic groups generated by hemoglobin degradation [5]. Other antimalarials used in ACT, for example, pyrimethamine and proguanil, inhibit the tetrahydrofolic acid cycle and thus eliminate an important cofactor for DNA synthesis. Despite the arsenal of drugs available for malaria treatment, the disease remains a worldwide public health problem. *P. falciparum* quickly develops resistance under selective drug pressure [5]. *P. vivax*, the most prevalent human parasite worldwide, has been shown to be resistant to chloroquine, including in Brazil [3]. Continuous efforts on the development of new antimalarials are required, and our primary method has been to use different approaches, such as testing natural products and synthetic molecules, as reviewed [6], [7].

Drug-resistant malaria parasites are believed to emerge through mutations in the active sites of drug targets [5] or from biochemical changes in the drug receptors [8]. The continued search for new molecular targets for drug design should broaden the therapeutic arsenal and strategies to fight drug resistance in human malaria. The lactate dehydrogenase enzyme from *P. falciparum* (*Pf*LDH) has been considered as a potential molecular drug target. Although the primary mechanism of action of quinoline drugs is by inhibition of heme polymerization [9], other molecular targets have been reported as being important for the improvement of their biological effectiveness against *P. falciparum*. Chloroquine interacts specifically with *Pf*LDH in the NADH binding pocket, occupying a position similar to that of the adenyl ring cofactor and hence acting as a competitive inhibitor for this critical glycolytic enzyme [9], [10], [11], [12].

The LDH enzyme catalyzes the interconversion of pyruvate to lactate in the final step of glycolysis, which is required for energy production in living cells. Ferriprotoporphyrin IX (hematin), one of the products of hemoglobin degradation by malarial parasites, intoxicates the parasite by competing with NADH for the active site of *Pf*LDH; parasite survival depends on polymerization of hematin to hemozoin, which remains active in the food vacuole of the parasite and causes parasite death [9]. The quinoline derivatives are believed to form complexes with the dimeric hematin, preventing the formation of hemozoin [11].

Analogs of NADH have been identified as new potential inhibitors to *Pf*LDH in DrugBank [13]. In previous docking studies, Molegro Virtual Docker software (MVD)® yielded higher docking accuracy than other docking programs; the accuracies were: MVD, 87%; Glide, 82%; Surflex, 75%; and FlexX, 58% [14]. A total of 50 compounds were selected based on their interactions with an active site similar to that of NADH; the three (itraconazole, atorvastatin and posaconazole) compounds that presented the best theoretical results were tested *in vitro* against *P. falciparum* blood parasites and against malaria in mice.

## Results

### Docking studies

**Figure 1:**
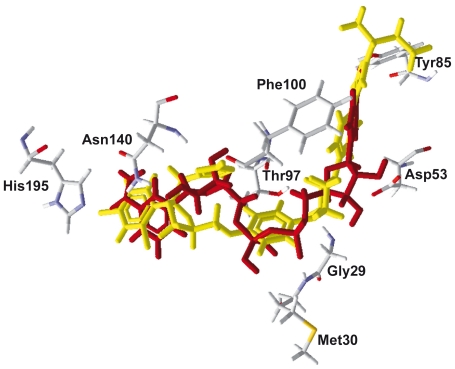
Superposition of the best conformation of itraconazole (in yellow) and NADH (in red) in the active site pocket of *P. falciparum* enzyme lactate dehydrogenase (*Pf*LDH).

The results of docking studies using the MolDock Scores observed for NADH and the 50 compounds chosen from the DrugBank [13] are summarized in Table 1. The superposition of NADH, as observed in the cavity of the crystallographic structure of *Pf*LDH, and the best conformation obtained theoretically for itraconazole are shown in Figure 1. This result suggests that the software reproduced the appropriate conformation of NADH inside its binding pocket in the *Pf*LDH active site.

**Table 1:** MolDock Scores observed for NADH and the 50 compounds chosen from the DrugBank [13].

Drugs	MolDock Score(kcal.mol^−1^)	Drugs	MolDock Score(kcal.mol^−1^)
NADH	−249.6	Cefotaxime	−143.2
Itraconazole	−218.5	Valaciclovin	−142.4
Atorvastatin	−209.3	AMP	−136.5
Posaconazole	−201.6	Cephapirin	−136.5
Rescinnamine	−201.0	Cefuroxime	−135.2
Cefpiramide	−195.0	Cefalotin	−132.9
Eprosartan	−177.0	Abacavir	−130.4
Cefditoren	−177.2	Entecavir	−130.4
Ergotamine	−175.0	Capecitabine	−129.1
Cefmenoxime	−173.3	Topotecan	−128.6
Dicloxacilin	−165.0	Tenoxican	−123.9
Novobiocin	−162.5	Genifloxacin	−120.6
Ceftriaxone	−157.4	Cefdinir	−117.2
Cefamandole	−154.9	Moxifloxacin	−116.4
Ceftazidine	−156.8	Clorafabine	−114.8
ATP	−155.2	Adenosine	−113.6
Cefamandole	−154.9	Zidovudine	−111.7
Dasatinib	−154.5	Nelarabine	−111.3
Droperidol	−152.3	Cladribine	−111.0
Cefixime	−151.0	Pentostanine	−110.7
S-Adenozylmetionine	−150.7	Vidarabine	−110.6
Paliperidol	−150.4	Trifluridine	−102.2
Rysperidone	−146.9	Zalcitabine	−94.4
Cefmetazole	−146.9	Gemcitabine	−92.1
Bethamidine	−144.3	Floxuridine	−91.4

The compounds atorvastatin and posaconazole also fitted well in the NADH pocket (data not shown), showing the best docking energy values, that is, closest to NADH (which has a docking energy of −249.6 kcal·mol^−1^). These three compounds were selected for further *in vitro* tests because they are commercially available for human use as well.

**Figure 2:**
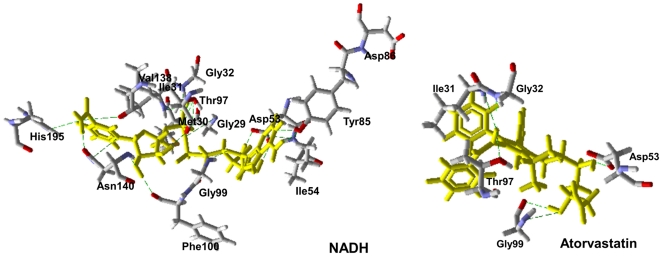
H-Bonds (in green) observed for NADH and atorvastatin (in yellow) with the active site residues of *Pf*LDH.

**Figure 3:**
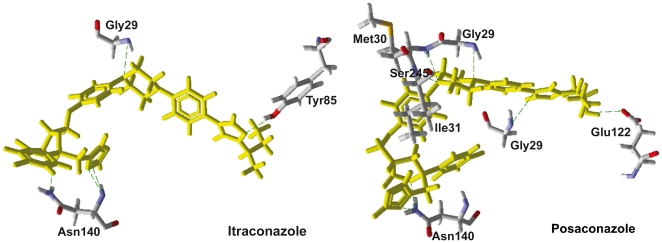
H-bonds (in green) observed for Itraconazole and Posaconazole (in yellow) with the active site residues of *Pf*LDH.

The active site residues that interact with NADH, itraconazole, atorvastatin and posaconazole inside *Pf*LDH are shown in Table 2, and the H-bonds between each compound and the *Pf*LDH active site are shown in Figures 2 and 3. The H-bond energy values were −1.9 kcal·mol^−1^, −5.0 kcal·mol^−1^ and −6.5 kcal·mol^−1^ for atorvastatin, itraconazole and posaconazole, respectively, and are all higher than that observed for NADH, which is able to make more H-bonds in the binding pocket than the studied compounds.

**Table 2:** Docking results for atorvastatin, posaconazole and itraconazole inside *Pf*LDH.

Drugs	MolDock Score (kcal.mol^−1^)	Hbond Score(kcal.mol^−1^)	Number of H-Bonds	Residues of the NADH binding site interacting with the ligands
NADH	−249.6	−29.3	22	Asn140, His195, Val138, Phe100, Gly99, Thr97, Gly32, Ile31, Met30, Gly29, Asp53, Ile54, Tyr85
Atorvastatin	−209.3	−1.9	6	Asp53, Thr97, Gly32, Ile31, Gly99
Itraconazole	−218.5	−5.0	5	Asn140, Gly29, Tyr85
Posaconazole	−201.6	−6.5	8	Gly99, Gly29, Met30, Ile31, Asn140, Ser 245, Glu122

### Activity of itraconazole, atorvastatin and posaconazole against *P. falciparum*

The *in vitro* tests against chloroquine-resistant *P. falciparum* clone W2 showed that itraconazole, atorvastatin and posaconazole were active. In two different immunoenzymatic assays (ELISA) with monoclonals anti-*Pf*LDH or anti-HRP2, the three compounds inhibited parasite growth at low doses. Posaconazole was the most active compound. Moreover, in one of the four experiments, the IC_50_ was 2.6 µM in the ELISA anti-*Pf*LDH; the average of IC_50_ from the experiments is shown in Table 3, corroborating our computer analysis and docking calculations (Table 1).

**Table 3:** *In vitro* activity of atorvastatin, itraconazole, posaconazole and chloroquine against *P. falciparum* as evaluated through their 50% inhibitory concentration (IC_50_) in immunoenzymatic assays (ELISA) performed with monoclonal antibodies against a parasite protein (*Pf*HRP2) or the enzyme lactate dehydrogenase (*Pf*LDH).

Drugs	IC_50_ (µM) Mean ± SD in ELISA tests and CQ ratio*			
	Anti-*Pf*LDH	CQ activity ratio**	Anti-HRP2	CQ activity ratio**
Atorvastatin	13.1±4.5	262	13.8±3.2	115
Itraconazole	9.3±0.8	186	9.2±1.6	77
Posaconazole	2.6±0.3	52	5.3±1.8	44
Chloroquine-CQ	0.05±0	-	0.12±0.09	-

*Average of 4 experiments.

**Ratio of chloroquine activity, significantly higher that of test compounds.

### Antimalarial tests in mice with malaria due to *P. berghei*

The compounds active *in vitro* were next tested in mice infected with *P. berghei*. Due to limitations in the availability of the purified compounds, they were first tested at doses of 20 mg/kg. All compounds inhibited parasite growth, especially atorvastatin and posaconazole, which reduced parasitemia by 41% and 46%, respectively, compared to untreated group (Table 4). Further tests confirmed the activity of atorvastatin (data not shown) and posaconazole. For the latter, a commercially available oral suspension of posaconazole, formulated for human use (Noxafil®) was acquired at a pharmacy in the USA and tested at 50 and 100 mg/kg. It reduced parasitemia in the treated mice by 45% and 71%, respectively, as compared to untreated controls (Table 4).

**Table 4:** Inhibition of *P. berghei* growth in mice infected with blood parasites that were then treated orally with atorvastatin, itraconazole, posaconazole or and chloroquine for three consecutive days in two independent experiments.

Drugs	Dose (mg/kg)	% Parasitemia Reduction*		Activity
		Exp. 1	Exp.2**	
Atorvastatin	20	41	40	Yes
Itraconazole	20	30	ND	Partial
Posaconazole	20	46	ND	Yes
	50	ND	45	Yes
	100	ND	71	Yes
Chloroquine	20	100	100	Yes

ND = not done.

*Reduction of parasitemia at day five of the experiment in relation to untreated controls (n = 4 to 6 mice per group).

**In the second experiment, posaconazole was diluted from a commercial oral suspension (Noxafil®).

Only the chloroquine-treated mice survived to day 30 post-inoculation; all the other mice died, despite the suppression of parasitemia in relation to the control mice.

However, itraconazole reduced parasitemia by only 30% whereas, as expected, chloroquine cured the animals at 20 mg/kg. By day 30, the last day of the trial, all chloroquine-treated mice were still alive and had negative blood smears for malaria parasites. Thus, this antimalarial is significantly more active than the tested drugs.

## Discussion

Although *Pf*LDH is not a direct chloroquine target, experimental data have shown that this enzyme binds to chloroquine [10], [12]. Based on this information, we studied 50 commercially available compounds as candidates to *Pf*LDH inhibitors. The compounds that presented the closest binding energy values to NADH, which we considered to be the best results, were itraconazole, atorvastatin and posaconazole. In our software simulation, these compounds also interacted with the residues present in the *Pf*LDH active site, suggesting a competitive inhibition with NADH. The selected compounds also presented strong stability inside the *Pf*LDH active site; thus, they could also dock in the NADH binding pocket of *Pf*LDH. This theoretical hypothesis proved correct in light of our experimental data from *in vitro* assays performed with *P. falciparum*. Indeed, the selected compounds, itraconazole, atorvastatin and posaconazole, were all active *in vitro*. The results with two different tests were similar: one used monoclonals specific to a *P. falciparum* parasite protein (HRP2) and the other used monoclonals against the *Pf*LHD enzyme.

Posaconazole, an inhibitor of ergosterol biosynthesis [15], was the most active compound against *P. falciparum*; it also was the most effective compound against murine malaria caused by *P. berghei*. Because posaconazole was the most promising compound *in vitro* and *in vivo* in the present antimalarial study, we conducted a second test using higher doses of the compound, this time in the form of a commercially available (USA) oral suspension for human use and confirmed its activity in mice. We hope to use this drug in subsequent human malaria trials. In other models, the *in vivo* activity of posaconazole depends on the interleukins IFN-γ and IL-12, such as in the case of *Trypanosoma cruzi* infections in mice [16]. Posaconazole has been considered a candidate for clinical trials in human Chagas disease caused by this hemoprotozoan parasite [15].

Itraconazole, acquired in tablet form and purified for the tests described herein, also caused a strong inhibition of *P. falciparum* growth *in vitro*; however, it was only partially active against *P. berghei* malaria in mice. The fact that the animals were not treated with the same pharmaceutical form (pellets) available for human use may explain its failure. Alternatively, it may have not been absorbed, may have been inactivated in the animal digestive tract or used in an insufficient dose. These possibilities should be further explored.

Atorvastatin, despite being over 100-fold less active than chloroquine *in vitro*, appears to be an attractive compound for the development of new antimalarials because its mechanism of action involves *Pf*LDH. In a recent work, atorvastatin activity was tested in combination with quinine and had a synergistic activity, enhancing antimalarial effects [17]. In addition, as recently shown, atorvastatin was able to reverse the binding of *P. falciparum* infected human erythrocytes (cytoadherence) to endothelial cells *in vitro* [18]. This drug is likely to become a good candidate for the treatment of severe malaria, specially if used together with other antimalarials. However, the ideal drug combinations and doses for human use are yet to be defined, either for ACT or with another antimalarial.

Itraconazole, like posaconazole and atorvastatin, does not require further testing for human toxicology and bioavailability because it is already approved and available for human use worldwide. Itraconazole is used against fungal infections and atorvastatin is the main component of the medicine Lipitor®, which is widely used to reduce cholesterol levels. Whether they can be useful for malaria treatment will depend on their possible synergisms with other antimalarials because only drug combination therapy is recommended for the control of malaria to avoid further selection for drug resistance. It would be desirable to perform further tests with the three compounds, applying them in different treatment routes and in ACT.

The *Plasmodium* lactate dehydrogenase (pLDH) enzymes found in all four species of human malaria parasites have been cloned, expressed and analyzed for structural and kinetic properties that may be explored for drug development. The pLDH from the species *P. vivax*, *P. malariae* and *P. ovale* exhibit 90–92% identity to *Pf*LDH. The catalytic residues and the cofactor sites are similar in the pLDH from *P. falciparum* and *P. malariae*, and the pLDH from *P. vivax* and *P. ovale* share one substitution. Homology modeling of the pLDH from *P. vivax*, *P. ovale* and *P. malariae* using the crystal structure of *Pf*LDH as a template yielded similar structures [19]. Thus, it would be desirable to have new anti-pLDH drugs that are effective against major species of human *Plasmodium* because cases of chloroquine-resistant *P. vivax* have already been reported [3], [20]. In addition, except for the catalytic residues (Arg171, Arg109, and the dyad His195/Asp168), *Pf*LDH has different active sites and substrate specificity loop residues than the human LDH isoforms (hLDH), reflecting a relative displacement of the nicotinamide ring and a volume increase of the active site compared with *Pf*LDH; *Pf*LDH displays kinetic differences with hLDH, suggesting that *Pf*LDH is a unique antimalarial target [19].

The study data suggest that the mechanism of parasite growth inhibition by the different compounds results from drug competition with NADH for the *Pf*LDH and that different methods used to measure drug activity against *P. falciparum in vitro* were equally efficient. The activity of itraconazole, atorvastatin and posaconazole, selected through docking studies and confirmed in biological assays, indicates that docking is an appropriate strategy for antimalarial discovery; furthermore, this technique is likely to be less expensive that traditional screening methods, especially because these compounds are commercially available and approved for human use. The best association(s) between these compounds and other antimalarials remains to be determined. Further improvements in the structures of the lead compounds could include additional pharmacophoric groups that can interact with more amino acids of the NADH pocket, leading to new and more effective antimalarials. Dynamic studies of these drugs binding with *Pf*LDH using more accurate methods can be used to evaluate the interactions between these drugs and the enzyme.

## Materials and Methods

### Molecular docking

The 3D structures of the *Pf*LDH complex with NADH and the substrate oxamate were obtained from the Protein Data Bank (PDB ID: 1LDG) [21]. For the docking studies, 50 compounds from DrugBank [13], all structural analogs of NADH, were selected using the search algorithm of the website and accessing the NADH chart in DrugBank through the option “show similar structures.” This option uses a locally developed simplified molecular input line entry specification (SMILES) string comparison method to identify related structures and perform structure similarity searches. All structures are converted into SMILES strings, and a substring-matching program (similar to BLAST) is used to identify similar structures. The scoring scheme is based simply on the number of character matches for the longest matching substring [13]. The selected compounds were submitted to docking studies using MVD® [14]. The candidates with the best conformational and energetic results were selected for further experimental tests. MVD® [14] was used to calculate the interaction energies between ligands and macromolecular systems from the 3D structures of the protein and ligands. The algorithm used was the MolDock Score, an adaptation of the Differential Evolution (DE) algorithm [14]; the MolDock Score energy, *E_score_*, is defined by Equation 1, where E*_inter_* is the ligand-protein interaction energy and E*_intra_* is the internal energy of the ligand. E*_inter_* is calculated according to Equation 2.
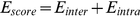
(1)

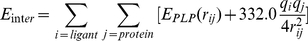
(2)


The E*_PLP_* term is a “piecewise linear potential” [22] that uses two different parameters, one for the approximation of the steric term (van der Waals) between atoms and another for the potential for hydrogen bonds; it describes the electrostatic interactions between charged atoms [14]. E_intra_ is calculated according to Equation 3.
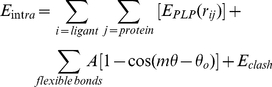
(3)


The first term in Equation 3 calculates all the energies involving pairs of atoms of the ligand, except those connected by two bonds. The second term represents the torsional energy, where θ is the torsional angle of the bond. The average of the torsional energy bond contributions is used if several torsions can be determined. The last term, E*_clash_*, assigns a penalty of 1,000 kcal·mol^−1^ if the distance between two heavy atoms (more than two bonds apart) is smaller than 2.0 Å, ignoring infeasible ligand conformations [14].

### Drug samples for pharmacological tests

Itraconazole [(2*R*,4*S*)-*rel*-1-(butan-2-yl)-4-{4-[4-(4-{[(2*R*,4*S*)-2-(2,4-dichlorophenyl)-2-(1*H*-1,2,4-triazol-1-lmethyl)-1,3-dioxolan-4-yl]methoxy}phenyl)piperazin-1-yl]phenyl}-4,5-dihydro-1*H*-1,2,4-triazol-5-one], which is commercially available as a generic >compound (Sporanox®, Janssen-Cilag) and is produced by Brainfarma, was purified into the crystallized form as previously described [23]. Atorvastatin [(3*R*,5*R*)-7-[2-(4-fluorophenyl)-3-phenyl-4-(phenylcarbamoyl)-5-(propan-2-yl)-1*H*-pyrrol-1-yl]-3,5-dihydroxyheptanoic acid] was provided by Farmanguinhos-FIOCRUZ as a purified compound. Posaconazole [4-(4-(4-(4-(((3r,5r)-5-(2,4-difluorophenyl)-5-(1,2,4-triazol-1-ylmethyl)oxolan-3-yl)methoxy)phenyl)piperazin-1-yl)phenyl)-2-((2s,3s)-2-hydroxypentan-3-yl)-1,2,4-triazol-3-one] was a kind gift from the Laboratory of Molecular Parasitology-FIOCRUZ, Belo Horizonte/MG, Brazil, where the compound has been studied against *T. cruzi* [16]. A second sample was later acquired in a drug store in USA to repeat the tests as Posaconazole oral suspension (Noxafil®) 200 mg/5 mL, Lot # 001R5H (Schering-Plough Research Institute, Kenilwort, New Jersey, USA).

All test drugs were assayed against *P. falciparum* diluted in dimethyl sulfoxide (DMSO 0.02% v/v) (Sigma-Aldrich, St. Louis, MO, EUA) from a 10 mg/mL stock solution and further diluted with RPMI 1640 (Sigma-Aldrich) supplemented with Hepes 25 mM (Sigma-Aldrich), sodium bicarbonate 21 mM (Sigma-Aldrich), glucose 11 mM (Sigma-Aldrich), glutamine 2% (Sigma-Aldrich) and gentamicin 40 mg/mL (Schering-Plough, Kenilworth, New Jersey, EUA). For each test well, the controls consisted of the parasite in culture without drug addition or with chloroquine at various concentrations.

### Continuous cultures of *P. falciparum*

The chloroquine-resistant and mefloquine-sensitive *P. falciparum* W2 clone [24] was maintained in continuous culture at 37°C in human erythrocytes (A^+^) in Petri dishes (Corning, Santa Clara, CA, USA) using the candle jar method [25] and grown in complete medium (RPMI 1640 supplemented with 10% human sera blood group A^+^), with daily medium changes. Parasite samples were also stored frozen in liquid nitrogen.

### Antimalarial tests *in vitro* against blood stages of *P. falciparum*

The effect of the test drugs against *P. falciparum* was determined from curves of inhibition of parasite growth *in vitro* as described previously [26] with some modifications [27]. Before the tests, the ring-stage parasites were concentrated in sorbitol-synchronized blood [28] and the suspension of infected red blood cells (iRBC) was adjusted for parasitemia and hematocrit following the specifications for each test; parasites were then distributed (180 µL/well) into a 96-well microtiter plate (Corning, Santa Clara, CA, EUA). All compounds were tested in triplicate for each dose in parallel with chloroquine, the standard antimalarial. The antiplasmodial activity was then measured using: (i) an ELISA anti-HRP2 test as previously described [29] and (ii) an ELISA anti-*Pf*LDH (*double-site enzyme-linked lactate dehydrogenase assay*) [30].

The effect of antimalarial drugs is initially characterized by the inhibition of parasite growth in drug-exposed cultures in comparison to a drug-free control culture. When performed using serial drug dilutions, sigmoid dose-response curves are generated and enable the determination of the 50% inhibitory concentration (IC_50_).

### ELISA anti-HRP2

The production of HRP2, a histidine- and alanine-rich protein, by *P. falciparum* parasites was tested *in vitro* as described previously [29]. Briefly, a suspension of iRBC with sorbitol-synchronized parasites, was adjusted to 0.05% parasitemia and 1.5% hematocrit, placed in 96-well plates containing the test and control drugs at various concentrations and incubated for 72 h under the culture conditions described above. After 24 h, the contents of the six control wells (parasites in drug-free medium) were harvested in microtubes and frozen for later use to further exclude the background value (i.e., the production of HRP2 during the first 24 h of incubation) by subtracting the average value obtained from these wells from the wells with the test and control drugs. After 72 h of incubation, the plates were frozen and thawed twice to lyse the erythrocytes.

To perform the test, a clean plate (Maxysorp, Nunc, Denmark) was first coated with 100 µL of the primary antibody anti-HRP2 (MPFM ICLLAB-55A®, USA) at 1.0 µg/mL. Following overnight incubation at 4°C, the monoclonal was discarded and replaced with 200 µL/well of the blocking solution PBS-BSA 2% (Phosphate Buffered Saline and Bovine Serum Albumin) (Sigma-Aldrich). Following a new incubation at room temperature for 2 h, the plate was washed three times with PBS/Tween20 at 0.05% (PBS-T). Then, each pretreated well received 100 µL of *P. falciparum* parasite culture (as described above), which was prehemolyzed by freeze-thawing at −70°C. In each test, two hemolyzed control sets of six wells each were used; one containing the 24 h cultures (background), the other with the 72 h parasite cultures. After incubation for 1 h at room temperature, the plate was again washed three times with PBS-T, incubated with 100 µL/well of the secondary antibody (MPFG55P ICLLAB®, USA), diluted 1∶5,000 times, and again incubated for 1 h at room temperature. After three more washes with PBS-T, each well received 100 µL of 3,3′,5,5′-Tetramethylbenzidine (TMB) chromogen (KPL, Gaithersburg, MD, EUA) and was incubated for 10 min at room temperature in the dark; the reaction was stopped with 50 µL/L of 1 M sulfuric acid and the absorbance was immediately read at 450 nm in a spectrophotometer (SpectraMax340PC^384^, Molecular Devices).

### ELISA anti-*Pf*LDH

The anti-*Pf*LDH test was performed as described previously [30]. Briefly, cultures of *P. falciparum* were adjusted to 0.5% parasitemia and 2% hematocrit, placed in 96-well plates with the test drugs or control antimalarial drugs at different concentrations. The plates were then incubated under the same culture conditions as described above for 48 h, frozen and thawed thrice to lyse erythrocytes.

To perform the anti-*Pf*LDH test, a clean plate was first coated with 100 µL/well of the primary antibody anti-*Pf*LDH (17E4 Vista Diagnostics International LLC®, USA) at 1.0 µg/mL. Following overnight incubation at 4°C, the monoclonal was then discarded and replaced with 300 µL/well of the blocking solution (PBS-BSA 1%); the plate was then incubated at room temperature for 4 h and washed four times with PBS-T. The precoated plate received 100 µL of the *P. falciparum* parasite cultures in each well (as above), which was prehemolyzed by freeze-thawing and diluted 1∶100 times in PBS/BSA 1%. For each test, two hemolyzed control sets of six wells each were used, one containing the parasite in cultures without drug addition and the other with uninfected red blood cells (RBCs).

The plate containing the parasite lysate and the monoclonal was again incubated for 1 h at room temperature, washed four times with PBS-T and then incubated with 100 µL/well of the secondary antibody (19g7, Vista Diagnostics International LLC®, USA) diluted 1∶5,000 times. After 1 h of incubation at room temperature and four washes with PBS-T, each well received 100 µL of streptavidin-HRP conjugate (Sigma-Aldrich) diluted 1∶1,000 and incubated for 30 min at room temperature. The plate was washed four times with PBS-T and incubated with 100 µl/well of TMB chromogen followed by 10 min of incubation at room temperature in the dark. The reaction was stopped with 50 µL/L of 1 M sulfuric acid and the absorbance was immediately read at 450 nm in a spectrophotometer (SpectraMax340PC^384^, Molecular Devices).

### Ethical committee approval for animal use

Methodological issues involving the use of laboratory animals in this study were approved by the Ethics Committee for Animal Use, the Oswaldo Cruz Foundation - Fiocruz (CEUA L-0046/08).

### Antimalarial tests in mice infected with *P. berghei*

The antimalarial chemotherapy suppressive tests were performed as previously described [31], with modifications [32]. The *P. berghei* NK65 strain, a chloroquine-sensitive parasite, was stored at −70°C and also maintained by weekly blood passages in outbred Swiss mice. For the chemotherapy experiments, adult female mice weighing 20±2 g were inoculated intraperitoneally with 1×10^5^ iRBC and kept together in a cage. Twenty-four hours after parasite inoculation, the mice were randomly distributed, six mice per cage, and then orally treated with the test and control compounds daily for three consecutive days. The drugs were freshly diluted with water, DMSO 3% or RPMI and given orally (200 µL per animal) at doses of 20–100 mg/kg. Posaconazole was used at a dose of 20 mg/kg body weight in the first experiment and at 50 and 100 mg/kg in the second experiment. Chloroquine-treated and untreated control groups were included in each test. Thin blood smears were taken starting at day five after parasite inoculation, Giemsa stained and examined microscopically. Drug activity was determined on the basis of the average parasitemia per group of mice. The percent inhibition of parasite multiplication in the treated groups was compared to the untreated controls and the parasite inhibition growth was calculated based on the percent parasitemia in the groups according to equation 4 [32].
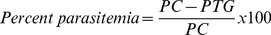
(4)


Where: PC is the parasitemia in the control group and PTG is the parasitemia in the test group. Drugs that reduced parasitemia by 29–40% were considered partially active; a reduction of >40% was considered active. Overall mortality was monitored daily until day 30 post-infection in all groups.

#### Supplementary Material

A table with the energy values of the main HBond interactions between residues of NADH binding site and NADH, atorvastatin, itraconazole and posaconazole is available as Table S1.

## Supporting Information

Table S1 HBond Energies between residues of NADH binding site and NADH, atorvastatin, itraconazole and posaconazole.
(DOC)
