# Supplementary material for: Antimalarial Activity of Potential Inhibitors of Plasmodium falciparum Lactate Dehydrogenase Enzyme Selected by Docking Studies
Source: PLoS One. 2011 Jul 14;6(7):e21237. doi: 10.1371/journal.pone.0021237 (PMC3136448; doi:10.1371/journal.pone.0021237)
Supplement: Table S1 — HBond Energies between residues of NADH binding site and NADH, atorvastatin, itraconazole and posaconazole. (DOC) [file pone.0021237.s001.doc]

Table S1 - HBond Energies between residues of NADH binding site and NADH, atorvastatin, itraconazole and posaconazole.

| **Drugs** | **Residues of the NADH binding site** | **HBond Energy**  **(kcal.mol^-1^)** |
| --- | --- | --- |
| NADH | Asn140 | -2.500  -1.931  -0.755 |
|  | His195 | -1.918 |
|  | Val138 | -1.059 |
|  | Phe100 | -2.500 |
|  | Gly99 | -1.611 |
|  | Thr97 | -2.500  -2.500 |
|  |  | -1.722  -1.052  -0.748 |
|  | Gly32 | -2.464 |
|  | Ile31 | -0.189 |
|  | Met30 | -2.488  -0.866 |
|  | Gly29 | -0.041 |
|  | Asp53 | -2.500  -2.500 |
|  | Ile54 | -0.620 |
|  | Tyr85 | -0.709  -2.500 |
| Atorvastatin | Asp53 | -2.500 |
|  | Thr97 | -0.415 |
|  | Gly32 | -1.857 |
|  | Ile31 | -0.915 |
|  | Gly99 | -1.901  -1.645 |
| Itraconazole | Asn140 | -1.870  -2.495  -1.566 |
|  | Gly29 | -0.470 |
|  | Tyr85 | -2.494 |
| Posaconazole | Gly99 | -0.633 |
|  | Gly29 | -0.556 |
|  | Met30 | -0.706 |
|  | Ile31 | -1.670 |
|  | Asn140 | -0.579  -1.057 |
|  | Ser245 | -2.476 |
|  | Glu122 | -2.168 |
|  |  |  |
